# Mechanisms of Resistance to CDK4/6 Inhibitors: Potential Implications and Biomarkers for Clinical Practice

**DOI:** 10.3389/fonc.2019.00666

**Published:** 2019-07-23

**Authors:** Amelia McCartney, Ilenia Migliaccio, Martina Bonechi, Chiara Biagioni, Dario Romagnoli, Francesca De Luca, Francesca Galardi, Emanuela Risi, Irene De Santo, Matteo Benelli, Luca Malorni, Angelo Di Leo

**Affiliations:** ^1^“Sandro Pitigliani” Department of Medical Oncology, Hospital of Prato, Prato, Italy; ^2^“Sandro Pitigliani” Translational Research Unit, Hospital of Prato, Prato, Italy; ^3^Bioinformatics Unit, Hospital of Prato, Prato, Italy

**Keywords:** CDK4/6 inhibitors, biomarker, thymidine kinase-1, PIK3CA, resistance, palbociclib, ribociclib, abemaciclib

## Abstract

The recent arrival of CDK4/6 inhibitor agents, with an approximate doubling of progression-free survival (PFS) associated with their use in hormone receptor-positive, HER2-negative advanced breast cancer (BC), has radically changed the approach to managing this disease. However, resistance to CDK4/6 inhibitors is considered a near-inevitability in most patients. Mechanisms of resistance to these agents are multifactorial, and research in this field is still evolving. Biomarkers with the ability to identify early resistance, or to predict the likelihood of successful treatment using CDK4/6 inhibitors are yet to be identified, and represent an area of unmet clinical need. Here we present selected mechanisms of resistance to CDK4/6 inhibitors, largely focussing on roles of Rb, cyclin E1, and the PIK3CA pathway, with discussion of associated biomarkers which have been investigated and applied in recent pre-clinical and clinical studies. These biological drivers may furthermore influence clinical treatment strategies adopted beyond CDK4/6 resistance.

In normal cell signaling, mitogenic signaling via pathways including ER and PI3K/Akt/mTOR activates the cyclin D1-cyclin-dependent kinases 4 and 6 (CDK4/6) complex. Once activated, CDK4/6 phosphorylates the retinoblastoma protein (Rb), an event which causes Rb to lose the ability to bind to the E2F family of transcription factors. E2F is therefore released, activating gene transcription and thus initiating progression of the cell cycle from G1 to S phase (resulting in DNA synthesis) (1). Dysregulation of the CDK4/6 pathway, occasioning unchecked cell-cycle progression and proliferation, has been implicated in breast cancer (BC) via various mechanisms, including amplification of cyclin D1 (2), gain of CDK4, low expression of p18/high expression of *RB1* (3) and inactivation of p16, the tumor suppressor protein and negative moderator of the cyclin D-CDK4/6 complex (4). The p16 protein, encoded by the *INK4a* gene, is a common target of inactivating mutations and deletions, operating upstream from RB (5).

The past decade has seen a radical shift in the management of advanced or metastatic hormone receptor-positive (HR+), human epidermal growth factor receptor-2 (HER2) negative BC. Endocrine therapy (ET) as monotherapy, previously thought to be the gold-standard in first and often subsequent lines of management, has been augmented and displaced in the treatment hierarchy by the emergence of selective, small-molecule inhibitors of CDK4/6. Three such agents are currently in widespread clinical use, with all three, when given in combination with ET, resulting in an approximate doubling of progression-free survival (PFS) in patients with advanced HR+/HER2–negative BC, compared to ET plus placebo. These consistent positive PFS results have been demonstrated in large phase III trials in the upfront setting [PALOMA-2 (6) for palbociclib plus letrozole; MONALEESA-2 (7) for ribociclib plus letrozole and MONARCH-3 (8) for abemaciclib plus letrozole or anastrozole], as well as in populations previously treated with ET for advanced disease [PALOMA-3 for palbociclib plus fulvestrant (9); MONARCH-2 for abemaciclib and fulvestrant (10); and MONALEESA-3 for ribociclib plus fulvestrant (11)]. However, *de novo* or acquired resistance to CDK4/6 inhibitors is an almost ubiquitous inevitability, which has stimulated substantial interest in examining potential mechanisms of resistance, ways to overcome it, and methods of identifying it. Recently published reviews offer detailed insight into the myriad of likely mechanisms of resistance (12–14); conversely, we focus primarily on selected mechanisms and associated biomarkers that are of particular clinical interest, of whose potential has been validated in recent studies.

## Mechanisms of Resistance—Avenues for Possible Molecular Biomarkers

### Loss of Retinoblastoma Susceptibility Gene Product (Rb) Function

Loss of Rb, the main target of CDK4/6, has been implicated by multiple preclinical studies in being a driver of resistance to CDK4/6 inhibitors (15–17). Without the inhibitory influence of Rb, transcription factors of the E2F family continue unchecked, thus facilitating unregulated cellular progression to S-phase entry. Conversely, higher levels of *RB1*, the gene encoding for Rb, and cyclin D1 (and lower levels of p16) are observed in human BC cell lines sensitive to palbociclib (18). Clinical evidence of acquired *RB1* mutations leading to CDK4/6 inhibitor resistance was recently reported in a case-series of three patients with metastatic BC who had genotyping performed in both tissue and blood samples before and after commencing a CDK4/6 inhibitor (19). Each somatic mutation was detected via ctDNA analyses performed at the point of disease progression, and was not present prior to initiation of CDK4/6 inhibition. Polyclonal *RB1* mutations were identified in patients assigned to the palbociclib arm of PALOMA-3, albeit at comparatively low frequency (4.7%) (20). RBsig, a gene expression signature of Rb loss-of-function, has been validated in identifying between palbociclib-sensitive and resistant BC cell lines (21), and has been associated with sensitivity to abemaciclib monotherapy in tumors derived from the neoMONARCH study (NCT02441948) (22). Similarly, a gene set containing E2F targets, the E2F regulon, was significantly associated with lack of PFS improvement from palbociclib combination in the PALOMA-3 trial (23). Of note, no interaction was found between treatment and RB1 expression in the same study, indicating that a wider analysis of RB pathway might be needed to identify resistant patients. Recent data emerging from genomic analysis of ER-positive BCs treated with CDK4/6 inhibitors revealed—not unsurprisingly—that loss of *RB1* was associated with treatment resistance. However, also implicated in resistance to CDK4/6 inhibition were loss-of-function mutations of *FAT1*, leading to cellular proliferation mediated via activation of the Hippo signaling pathway and elevations in CDK6, thus revealing an additional and intriguing potential mechanism of resistance (24).

### Cyclin E1 (CCNE1)

*CCNE1*, the gene that encodes cyclin E1, is upregulated in models with resistance to CDK4/6 inhibitors (25, 26). Data emerging from PALOMA-3 suggests that *CCNE1* expression is associated with benefit from palbociclib (23), in line with previous pre-clinical data which suggested *CCNE1* amplification is associated with acquired resistance to palbociclib (27), as well as exploratory data derived in the neoadjuvant NeoPalAna trial, associating high levels of *CCNE1* with palbociclib resistance (28). Tumor tissue procured from recurrent disease on trial was assessed via mRNA profiling, assessing a range of cell cycle-related genes. Although all biomarker groups derived benefit from palbociclib, those with low tumor *CCNE1* expression had a greater response (median PFS for those receiving palbociclib plus fulvestrant, 14.1 months; vs. 4.8 months in those receiving fulvestrant plus placebo) than those with high *CCNE1* expression (7.6 months vs. 4.0; palbociclib plus fulvestrant vs. fulvestrant plus placebo, respectively). The predictive power of *CCNE1* mRNA was stronger in metastatic biopsies (interaction *p* < 0.001) than archived primary biopsy samples (interaction *p* = 0.09). Investigators provided further validation in an independent cohort (*N* = 61) drawn from the Preoperative Palbociclib (POP) Clinical Trial (NCT02008734), wherein high *CCNE1* mRNA expression correlated with a significantly lower anti-proliferative response to palbociclib. Contrastingly, no such association with *CCNE1* expression and PFS was found in the biomarker analyses of MONALEESA-2, with near-identical hazard ratios observed across expression groups (HR 0.54 95% CI 0.38–0.78 for *CCNE1* high expression; HR 0.53 95% CI 0.34–0.83 for low expression) (29). An earlier analysis of tumor specimens from PALOMA-2 also failed to demonstrate an association between *CCNE1* expression and benefit from palbociclib (30).

### Combining Rb and CCNE1—Possible Biomarker of CDK4/6 Sensitivity

A recent analysis of cell cycle-related markers found in a large panel of HR+ BC cell lines was recently reported, describing findings identified by gene-expression profiles and western blot (31). Both modalities identified that concurrent overexpression of *CCNE1* and down-regulation of Rb occurred at the time of palbociclib resistance. Subsequent *in silico* analyses, correlating the ratio between *CCNE1* and *RB1* expression levels (CCNE1/RB1) with palbociclib IC50 in a large dataset of cell lines, showed the ratio outperformed both *CCNE1* and/or *RB1* when they were utilized as sole markers. Furthermore, retrospective analyses showed CCNE1/RB1 to be an adverse prognostic factor, with the ability to differentiate between palbociclib sensitive and resistant patients enrolled in the neoadjuvant NeoPalAna trial (28).

### Initially Promising Targets but Negative Data: Cyclin D and p16

*CCND1*, the gene coding for cyclin D1 is amplified in approximately 15% of all BCs, and overexpression of cyclin D1 is observed in around 50% (32). Given the crucial role cyclin D1 plays in cell cycle mediation and its interplay with CDK4/6, it has been hypothesized that expression levels or dysregulation of cyclin D1 may relate to response to CDK4/6 inhibition. Similarly, intuitively, loss of p16^INK4A^ and the consequent deficit of its usual inhibitory action on cyclin D1 would appear to be a reasonable premise of CDK4/6 inhibitor resistance. However, this is largely not been borne out in the clinical setting. In a phase II study of single-agent palbociclib, low p16 did not correlate with clinical outcome in Rb-positive, heavily pre-treated advanced BC (33). In the same study, amplification of cyclin D1 was also not associated with clinical benefit or PFS. PALOMA-1 failed to show any significant difference in PFS in patients whose tumors harbored evidence of a loss of p16 or *CCND1* amplification, compared to unselected patients (34). Expression levels of cyclin D1 was not associated with benefit from palbociclib in PALOMA-3 (23).

### PIK3CA—Activation of Growth Factor Signaling Pathways

Mutations in the phosphatidylinositol 3-kinase (PI3K) catalytic subunit (PIK3CA) are found in approximately 40% estrogen receptor-positive BC (3). The PI3K/mTOR pathway has been shown to be upregulated in response to chronic exposure to CDK4/6 inhibitors, which in turn upregulates cyclin D. In the absence of CDK4 and CDK6, activated cyclin D can activate CDK2, which subsequently drives cell cycle progression (27). Circulating tumor DNA sequencing was performed on 195 patients enrolled in the PALOMA-3 study, comparing baseline and end-of-treatment analyses (20), demonstrating the emergence of driver mutations in PIK3CA and ESR1. Patients with a history of greater drug exposure appeared more likely to develop driver gene mutations, perhaps underlining the role that drug pressure plays in clonal expansion. Contrastingly, PIK3CA mutations were detected in the circulating DNA of 129 patients enrolled in PALOMA-3, with no significant association observed with response to treatment (9). Similarly, biomarker analysis of MONALEESA-3 demonstrated consistent benefit from ribociclib plus fulvestrant, irrespective of PIK3CA alteration status, as detected in baseline circulating tumor DNA (35). Functioning downstream of PI3K is 3-phosphoinositide-dependent protein kinase 1 (PDK1), a vital requisite for the full activation of AKT (36). The PI3K-PDK1 signaling pathway has been implicated in mediating resistance to CDK4/6 inhibitors, with ribociclib-resistant BC cell lines demonstrating an increase in PDK1 levels following drug exposure, resulting in activation of the AKT pathway (37).

## Prediction of Sensitivity or Early Response to CDK4/6 Inhibition—A Possible Role for TK1

Thymidine kinase-1 (TK1) is an enzyme in the pyrimidine salvage pathway that plays a critical role in the synthesis of DNA and in cell proliferation (38). High TK1 levels and activity in primary BC tissue correlate with poor prognosis (39, 40). Malignant cells can secrete pathological levels of TK1 detectable in blood, whereas in disease-free controls, levels are low or undetectable (41), with similar patterns reported in membrane expression of TK1 (42). TK1 as a marker of cell proliferation has been known and studied for some decades, but until recently, widespread, reliable quantification of absolute levels and activity have been limited, with most historical tests being radioimmunoassay-based. DiviTum (Biovica International, Sweden) is a refined ELISA-based assay capable of estimating TK1 activity (TKa) in cell lines, plasma and serum. Previous studies have suggested baseline and repeated assessments of TKa during the course of treatment may provide prognostic information (43–45).

### TK1 as a Biomarker—Founding Data Within Endocrine Therapy Studies

Previous studies have validated the use of DiviTum, both as a prognostic marker, and as one of response to ET. A pilot study of 31 women with advanced HR+/HER2 negative BC commencing on a new line of palliative endocrine therapy showed that those with low baseline levels of plasma TKa had a median PFS of 25.9 months, vs. 5.9 months in those with high baseline levels (*p* = 0.012) (46). Furthermore, patients whose TKa levels dropped after 1 month of ET demonstrated a significantly higher median PFS than those in whom TKa levels increased on treatment (14.5 months vs. 3.8, respectively; *p* = 0.0026).

These findings were upheld by a second retrospective study of a larger, more heavily pre-treated population derived from the cohort of EFECT, a landmark study which originally compared head-to-head palliative exemestane vs. fulvestrant (47). Again, baseline TKa levels proved prognostic: patients with low baseline readings had a median TTP (mTPP) of 5.03 vs. 2.57 in those with high baseline readings (*p* < 0.001). Patients whose TKa increased from baseline after 3 months of treatment had a significantly shorter mTTP (3.39 months, 95% CI: 2.14–4.11) than those whose TKa did not increase (5.39 months, 95% CI: 4.01–6.68) (*P* = 0.0045). After adjusting for major prognostic factors, TKa remained an independent marker (48).

### TK1 and CDK4/6 Inhibitors

Evidence of the prognostic role TK1 may play in HR+ BC patients has provided a proof-of-concept to justify moving investigation forward into the field of CDK4/6 inhibitors. Recent data suggests potential utility for TKa as a marker of CDK4/6 inhibition in patients receiving neoadjuvant ET plus palbociclib (49). DiviTum was employed in an analysis of serum samples derived from NeoPalAna, a neoadjuvant trial of 4 weeks of anastrozole monotherapy followed by four cycles of additional palbociclib, followed by a subsequent palbociclib washout in all but eight patients (28, 49). TKa was shown to markedly reduce with the introduction of palbociclib, rising at washout (but remaining suppressed in patients who did not receive washout). There was high concordance between changes in TKa and tumor Ki67 in the same direction from baseline to C1D15 and from C1D15 to point of curative surgery. This led to the conjecture that TKa may be seen as a dynamic marker that signifies the presence or absence of palbociclib activity. However, there is some pre-clinical evidence suggesting that TKa may precede a significant reduction in cellular proliferation. In a panel of HR+ BC models—both with sensitivity to palbociclib, and with acquired resistance to the drug—exposure to escalating levels of palbociclib and its relation to cellular proliferation and TKa was examined (50). In palbociclib-sensitive models, TKa significantly reduced after 3 days of drug exposure compared to control (*p* < 0.05). Concurrently, cellular proliferation (as assessed by methylene blue assay) was observed to drop significantly after a minimum of 6 days, suggesting TKa may be an early marker of proliferative inhibition in response to palbociclib. This phenomenon was not observed in models with acquired resistance to palbociclib. The prognostic ability of TKa has been clinically validated in planned translational studies of plasma derived from the TREnd study (NCT02549430), a phase II trial which tested the activity and safety of single-agent palbociclib against palbociclib combined with the ET the patient had received (and progressed on) most recently before enrollment (51). Not unlike to previous findings in ET-based TK studies, TREnd patients with a low baseline TKa had a significantly longer PFS compared to those whose levels were high at study commencement. Similarly, on treatment, those patients whose TKa levels rose had a shorter time to disease progression compared to those patients whose levels remained stable or dropped in response to treatment (52). The prognostic role of serum TK1 assessed at baseline and on treatment is being further explored in two ongoing clinical trials of luminal BC patients treated with CDK4/6 inhibitors and ET: BIOITALEE (NCT03439046), a Phase 3b biomarker study of ribociclib plus letrozole in the first-line setting and PYTHIA (NCT02536742), a phase 2 biomarker discovery trial of palbociclib and fulvestrant in patients with endocrine resistant disease.

## Implications on Therapeutic Approaches Following Progression on CDK4/6 Inhibitors

### Primary Resistance to CDK4/6 Inhibitors

Approximately 10% of patients will have primary resistance to CDK4/6 inhibitors. Biomarkers may have future potential to identify such patients at baseline or soon after commencing treatment, thus facilitating an early switch to a more efficacious treatment. For instance, patients with evidence of functional Rb loss at baseline are not likely to benefit from CDK4/6 inhibition. Similarly, baseline evidence of increased cyclin E1 expression, or the CCNE1/RB ratio may also play a role in identifying these patients. Peripheral evidence of ongoing neoplastic proliferation, as manifested by a rise in TK1 activity within a month of commencing therapy, may also provide a marker of early resistance.

### Secondary Resistance to CDK4/6 Inhibitors

An unanswered question regards the continuation of CDK4/6 inhibitors beyond progression on these agents. The premise that continuing a CDK4/6 inhibitor beyond progression may prove an effective strategy is being tested by several ongoing Phase 1 and 2 trials (MAINTAIN NCT02632045, PACE NCT03147287, NCT01857193, NCT 02871791, and TRINITI-1 NCT 02732119). Mutations in *RB1*, resulting in activation of other cell cycle factors, such as E2F and the Cyclin E-CDK2 axis, has been demonstrated in cases of acquired resistance (19, 53). This in turn results in independence from the CDK4/6 pathway for cell cycle progression from G1 to S phase. In such cases, in the setting of disease progression on a CDK4/6 inhibitor, concurrent biomarker evidence of a functional loss of Rb may support a switch to a new agent, rather than continuing CDK4/6 agents beyond progression.

### A Potential Role for PIK3CA Inhibitors?

PI3K-dependent activation of non-canonical cyclin D1-CDK2 and resultant recovery of Rb phosphorylation and S phase entry has been implicated in early resistance to CDK4/6 inhibition, with combined PI3K and CDK4/6 inhibition demonstrating the ability to overcome resistance to CDK4/6 inhibitors in BC cell lines (27). Hence, the role that PIK3CA inhibitors may play in overcoming resistance is of relevant interest. The SOLAR-1 trial randomly assigned 572 patients with pre-treated HR+/HER2–negative advanced BC to receive the oral PIK3CA inhibitor alpelisib plus fulvestrant or fulvestrant plus placebo (54). The primary endpoint was PFS in patients with PIK3CA mutations detectable in tumor tissue (*n* = 341). After a median follow up of 20 months, the median PFS was almost double in mutation-positive patients receiving alpelisib compared to those receiving placebo (11.0 months vs. 5.7 months, respectively; HR 0.65 95% CI 0.50–1.25 *p* = 0.00065). Further data, reporting the efficacy of alpelisib according to mutational status evaluated by ctDNA, suggested an even greater clinical benefit than tissue analysis (55). In patients with a PIK3CA mutation detected via liquid biopsy (*n* = 186), there was a 45% risk reduction in PFS (HR 0.55 95% CI 0.39–0.79). In the small number of patients who had previously received CDK4/6 inhibition (*n* = 20), there was a 52% risk reduction in PFS in favor of alpelisib over placebo (HR 0.48 95% CI 0.17–1.36). Alpelisib is selective for the alpha isoform of PI3K, which has so far set it apart from pan-PI3K inhibitors, which have reported notably poor safety profiles (56, 57). Nevertheless, data from SOLAR-1 still reflect considerable toxicity. All-grade hyperglycaemia occurred in 64% of patients receiving alpelisib (37% occurring at grade 3/4), 58% reported diarrhea, 45% nausea and 36% developed rash (10% at grade 3/4). Five percent of patients discontinued from the alpelisib arm due to adverse events (54).

Whilst the number of patients with prior exposure to CDK4/6 inhibitors subsequently enrolled in SOLAR-1 was small, it is not unreasonable to consider—in patients harboring an actionable mutation—PIK3CA inhibition following disease progression on a CDK4/6 agent. This is particularly relevant, given the likelihood of emergence of driver mutations of PIK3CA secondary to previous ET and CDK4/6-targeted therapies (20). Whilst some pre-clinical data suggest that triplet therapy, combining ET plus CDK4/6 inhibitors with PIK3CA agents may be better in preventing acquired CDK4/6 resistance than doublet regimens (27), this approach may potentially come at the cost of increased toxicity (58). Clinical trials investigating the feasibility and utility of combining CDK4/6 and PI3K inhibition are ongoing (Table 1). Another alternative may be to expose patients to these agents sequentially rather than simultaneously, reserving PIK3CA inhibition for those harboring a druggable mutation following exposure to CDK4/6 inhibition. This tactic is being tested in a currently-recruiting phase II study, which will assess the efficacy and safety of combining alpelisib and ET in patients with PIK3CA mutations whose disease has progressed on or after receiving a CDK4/6 inhibitor plus ET (NCT03056755). A summary of clinical trials of PIK3CA agents, currently recruiting patients with endocrine receptor-positive advanced BC, is presented in Table 2.

**Table 1:** Ongoing trials evaluating the combination of CDK4/6 inhibitors with PIK3CA agents in breast cancer [ClinicalTrials.gov June 2019].

**ClinicalTrials.gov identifier**	**Phase**	**Setting/key eligibility**	**Intervention**	**Primary endpoint(s)**
NCT02088684	Phase 1b/2* (*Phase 2 portion of study not opened due to sponsor decision)	ER+/HER2–negative metastatic or advanced disease	Ribociclib + fulvestrant + buparlisib *or* ribociclib + fulvestrant + alpelisib *or* ribociclib + fulvestrant	DLT (Phase 1b part only)
NCT02389842	Phase 1b (dose escalation and expansion phases)	Specific sub-group for patients with PIK3CA mutation	Palbociclib + taselisib + fulvestrant *or* palbociclib + taselisib *or* letrozole + palbociclib + taselisib	Recommended Phase 2 dose Safety and toxicity Anti-tumor response
NCT03939897	Phase 1/2	Endocrine resistant metastatic disease	Abemaciclib + fulvestrant + copanlisib vs. abemaciclib + fulvestrant	DLT PFS
NCT02684032	Phase 1b (dose escalation and expansion phases)	ER+/HER2 negative metastatic disease	Letrozole + palbociclib + gedatolisib *or* palbociclib + fulvestrant + gedatolisib	DLT rate ORR
NCT03128619	Phase 1/2	Locally advanced or metastatic disease not previously treated in advanced setting (Phase 1b portion); Stage I, II or III disease (Phase 2 (portion)	Copanlisib D1,8,15 + letrozole continuously *or* copanlisib D1,8,15 + palbociclib + letrozole continuously *or* palbociclib + letrozole for 14 days, followed by copanlisib D 1,8,15 + letrozole continuously	MTD Change in Ki67 on treatment DLT rate
NCT02154776	Phase 1 (dose escalation, open label)	ER+/HER2–negative advanced disease	Ribociclib + buparlisib + letrozole	DLT rate Safety and tolerability

*DLT, dose limiting toxicity; ER+, endocrine receptor-positive; MTD, maximum tolerated dose; ORR, objective response rate; PFS, progression-free survival*.

**Table 2:** Currently enrolling trials recruiting patients with hormone receptor-positive metastatic breast cancer, evaluating the role of PIK3CA agents [ClinicalTrials.gov June 2019].

**ClinicalTrials.gov identifier**	**Phase**	**Setting/key eligibility**	**Intervention**	**Primary endpoint(s)**
NCT03006172	Phase 1 Dose escalation and expansion stages	PIK3CA mutation-positive	GDC-077 as single agent and also in combination with ET and CDK4/6i	% of pts with DLTs Recommended Phase 2 dose % of pts with AEs and SAEs
NCT03207529	Phase 1 Dose escalation and expansion stages	AR-positive and PTEN positive metastatic disease	Alpelisib + enzalutamide	MTD
NCT02705859	Phase 1b/2 Single arm	HER2-positive disease	Copanlisib and trastuzumab	MTD with trastuzumab CBR
NCT03767335	Phase 1b Dose escalation and expansion stages	HER2-positive disease (+/– HR-positivity)	MEN1611 + trastuzumab +/– fulvestrant (in HR-positive disease)	MTD Recommended Phase 2 dose
NCT03386162	Randomized Phase 2	PIK3CA mutation-positive Patients not progressing after 6–8 cycles of 1st or 2nd line chemotherapy	Fulvestrant + alpelisib vs. maintenance chemotherapy	PFS
NCT03056755	Phase 2 Non-comparative study	PIK3CA mutation-positive Post progression on CDK4/6i	Alpelisib + fulvestrant OR Alpelisib + letrozole	% of patients alive without progressive disease (at ~6 month mark)
NCT03337724	Phase 3 Randomized, double blind	PIK3CA/AKT1/PTEN-altered disease Accepting TNBC or HR+/HER2neg	Ipatasertib + paclitaxel vs. placebo+paclitaxel	PFS

*AE, adverse event; AKT1, Alpha serine/threonine protein kinase; CBR, clinical benefit rate; CDK4/6i, Cyclin-dependent kinase 4/6 inhibitor; DLT, dose-limiting toxicity; HR, hormone receptor; ET, endocrine therapy; MTD, maximum tolerated dose; PIK3CA, phosphatidylinositol 3-kinase catalytic subunit; PFS, progression-free survival; PTEN, phosphate and tensin homolog tumor suppressor; SAE, serious adverse event; TNBC, triple negative breast cancer*.

## Author Contributions

All authors listed have made a substantial, direct and intellectual contribution to the work, and approved it for publication.

### Conflict of Interest Statement

AD has received honoraria from, and served as an advisory board member for, Pfizer, Lilly, AstraZeneca, and Novartis. AD has received research funding from Pfizer, AstraZeneca, and Novartis. LM has received consultation fees from Novartis and research support from Pfizer. The remaining authors declare that the research was conducted in the absence of any commercial or financial relationships that could be construed as a potential conflict of interest.
